# A High-Resolution Digital Pathological Image Staining Style Transfer Model Based on Gradient Guidance

**DOI:** 10.3390/bioengineering12020187

**Published:** 2025-02-16

**Authors:** Yutao Tang, Yuanpin Zhou, Siyu Zhang, Yao Lu

**Affiliations:** 1 School of Computer Science and Engineering, Sun-Yat sen University, Guangzhou 510006, China; tangyt3@mail2.sysu.edu.cn (Y.T.); zhouyp6@mail2.sysu.edu.cn (Y.Z.); 2 Vertex Pharmaceuticals, 50 Northern Avenue, Boston, MA 02210, USA; zhsiyu001@gmail.com

**Keywords:** pathological image, staining style transfer, deep learning, gradient guidance

## Abstract

Digital pathology images have long been regarded as the gold standard for cancer diagnosis in clinical medicine. A highly generalized digital pathological image diagnosis system can provide strong support for cancer diagnosis, help to improve the diagnostic efficiency and accuracy of doctors, and has important research value. The whole slide image of different centers can lead to very large staining differences due to different scanners and dyes, which pose a challenge to the generalization performance of the model application in multi-center data testing. In order to achieve the normalization of multi-center data, this paper proposes a style transfer algorithm based on an adversarial generative network for high-resolution images. The gradient-guided dye migration model proposed in this paper introduces a gradient-enhanced regularized term in the loss function design of the algorithm. A style transfer algorithm was applied to the source data, and the diagnostic performance of the multi-example learning model based on the domain data was significantly improved by validation in the pathological image datasets of two centers. The proposed method improved the AUC of the best classification model from 0.8856 to 0.9243, and another set of experiments improved the AUC from 0.8012 to 0.8313.

## 1. Introduction

Cancer is a prevalent cause of human death and affects the quality of life in the world today [1]. In most countries, cancer is the leading or second leading cause of death in people under 70 years of age [1]. The results of the last two global cancer statistics (2018 and 2020) show that the prevalence and mortality of most cancers are increasing [1]. Pathologic diagnosis is the gold standard for cancer diagnosis and plays an important role in cancer screening, diagnosis, and treatment. In clinical practice, pathologists perform histological examinations of pathological tissue sections under different magnifications and give diagnosis opinions. Digital pathology technology combined with a digital imaging system and traditional optical imaging system can provide doctors with higher resolution, easier to save, manage, transmit, and browse images, that are more conducive to remote consultation and pathology teaching. Doctors can perform histological examinations of pathological tissue sections on high-resolution displays through a computer and can consult with specialists in other hospitals through the Internet. The digitization of pathological tissue sections is also more convenient for medical staff to save and organize.

However, pathological diagnosis is still a time-consuming and intensive work, which requires repeated observation by pathologists. From the beginning to the completion of diagnosis and evaluation, the work undertaken often requires a lot of time and energy. The growing demand for pathological examinations and the serious shortage of pathologists have aggravated this situation. Pathologists often suffer from varying degrees of eye fatigue during clinical work, which leads to misdiagnosis and missed diagnosis [2,3]. Therefore, researchers hope that Computer-Aided Diagnostics (CADs) can free doctors from repetitive reading and diagnosis work and alleviate the imbalance of medical resources supply and demand. At present, CAD systems combined with deep learning have made a lot of progress in digital pathology, and some progress has been made in the tasks of the benign and malignant diagnosis of digital pathological images [4], cancer subtype classification [5], and the survival analysis of patients after surgery [6].

In digital pathological diagnosis, deep learning is a representation learning approach that has proven to be suitable for solving image analysis challenges in the context of color normalization, detection, segmentation, feature extraction, and classification [7,8,9,10,11]. In addition to these remarkable functional features, decision support systems based on deep learning are expected to reduce the workload of clinicians and improve the accuracy and efficiency of pathological interpretation [12,13,14,15]. However, many unique features of pathology limit the development of powerful deep learning models and the application of this promising cutting-edge technique in clinical practice. The lack of fixed standards for processing digital pathology images, such as digital imaging and communication in medicine, makes it difficult to obtain large digital pathology image sets with good structure in multiple centers. In addition, staining is a preprocessing step for histopathological tissue and is associated with significant heterogeneity at both intra- and inter-institutional levels. In the preprocessing step, staining protocols between different full-slide scanners, devices, dye manufacturers, and institutions, as well as biological differences between patients, can lead to differences in the color of digital pathology images [16]. Although these issues do not interfere with the pathological interpretation performed by expert pathologists, the consequent inconsistency may pose a great challenge to the generalization performance of deep learning models [17].

In order to overcome the heterogeneity of stained slides, there have been many studies designed a variety of color normalization methods to reduce the effect of color changes, which can be mainly divided into four categories [18,19]. First, methods based on global color matching, including histogram specification [20] and the Reinhard method [21], convert images from RGB to LAB color space. However, in these methods, the staining is not properly separated and some artifacts are introduced [22]. Second, supervised stain separation techniques, such as those reported in the study by Macenko et al. [23,24], separate stains by working at the pixel level. However, they require prior information in the training set and are not suitable for computer-aided diagnosis systems. Moreover, in these methods, the structural information of the source image is not preserved [22]. Third, the use of the unsupervised stain separation methods of Vahadane et al., such as complete color normalization [25] and structure preserving color normalization (SPCN) [26]. These methods do not require prior information and preserve the structure of the original image. However, they do not retain the full color information of the source image. Fourth, the use of neural network approaches including those using sparse autoencoders [27] and those based on unpaired image-to-image transformation using cycle-consistent generative adversarial networks (CycleGAN) [28]. Compared to other traditional color normalization methods, deep learning methods using CycleGAN show excellent visual similarity between image domains both quantitatively and qualitatively, and because they learn the entire distribution of images [16], representative reference images are not manually prepared. In particular, CycleGAN as a style transfer technique [29] may be a promising strategy to overcome poor model performance when dealing with external images. Finally, data augmentation in the training phase has also been explored to expand data diversity [30]. However, due to the simplicity of this approach, its application to many stained tissues in real-world settings is obscured, and it turns out to be insufficient for increasing the likelihood of model generalization [31].

With the advances in visualization technology and artificial intelligence (AI), the development of tumor analysis algorithms based on pathological section images and the support of computer-aided interpretation by pathologists is still an active research area [32]. As we mentioned earlier, due to the lack of unified standards for current digital pathology technology, the staining reagents and electronic scanners used by various medical centers are not the same, which may lead to a large gap in the staining of digital pathology images in different medical centers. Studies have shown that the visual differences in pathological images directly affect the quality and accuracy of pathological diagnosis [33], and the difference in color domain between images is also a major factor affecting the generalization of the model. In order to solve the problem of model performance degradation caused by staining differences between digital pathology images from different centers, existing studies mainly proceed using the following four aspects:

(1) By using only grayscale images to train and test the model, the problem caused by color differences is avoided. Although this method enhances the generalization of the model to a certain extent, it also reduces the classification performance of the model itself.
(2) Data augmentation based on staining is performed on digital pathological images to enable the model to learn more potential staining differences during training. This method forces the model to learn more texture-based features, but a lot of information in digital pathological images is reflected through staining, so this method may also enhance the generalization while reducing the performance of the model [34].
(3) All pathological images are matched to a fixed color combination by preprocessing. This method may lead to the introduction of artifacts at the pretreatment stage because the staining is not properly separated. Moreover, this method requires a lot of prior knowledge and is not suitable for CAD systems.
(4) The staining pattern of test data is transferred to the staining pattern of training data through the dye transfer algorithm, so that the test data can be tested under the original model after the dye transfer [16,28]. This method does not need to change the original trained model, so it does not degrade the performance of the model. Simultaneous dye transfer algorithms are usually based on unpaired generative adversative networks, which do not require additional labeling and are very easy to deploy. Therefore, this attempt has attracted wide attention from researchers.

**Figure 1:**
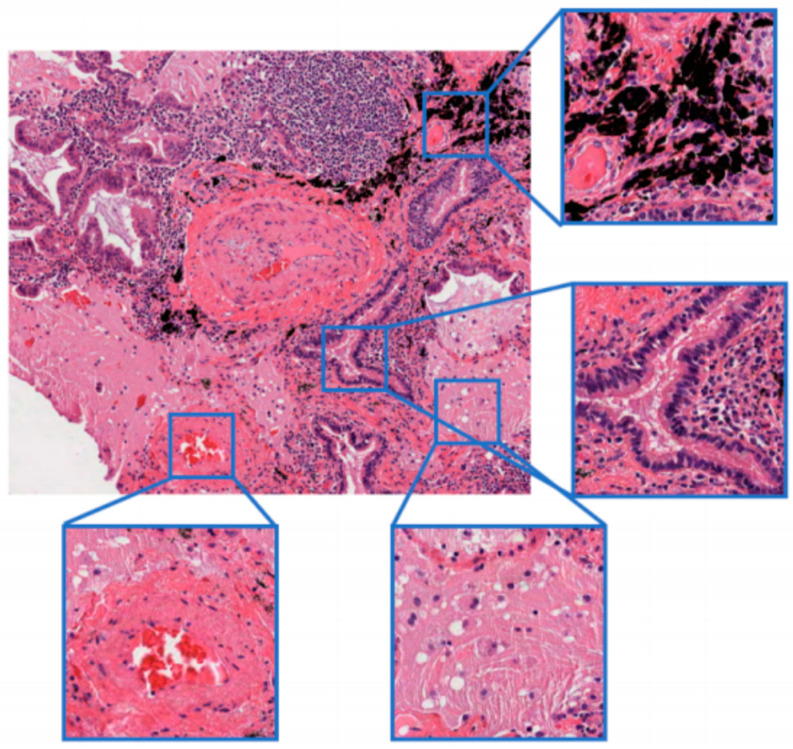
Color differences within a WSI. Even if different squares are taken in the same WSI, there is a huge difference in the color between these squares.

Although the dye transfer method is helpful to enhance the generalization performance of the model under multi-center testing, there are still the following challenges in this study:

(1) The resolution of the whole slide image (WSI) is very high, and the color differences in different tissue structures in a single WSI are observed. If the color transfer algorithm is trained at a higher resolution, the trained model may have instability or low contrast. As shown in Figure 1, if the picture of small squares is captured at a higher resolution, the color difference within different small squares in the picture is huge. When we use the unpaired generation algorithm for stain transfer, the cube images of the test data may match to any of the cube images shown in Figure 1.
(2) The diagnostic model of benign and malignant digital pathological images depends on the high resolution of WSIs. If the color transfer algorithm is trained under low-resolution WSIs, although the small blocks of the same size contain a larger scale of cell tissues at low resolution, which can alleviate the problem caused by the color difference within the WSI, the low-resolution WSIs will cause a degradation of the model performance.
(3) The regularity of the nuclear edge and cell membrane edge is very important for the diagnosis of benign and malignant cells, so the color migration algorithm should ensure that the edges of the nucleus and cell membrane are as clear as possible based on the premise of correct migration and staining.

In order to address the above challenges and solve the contradiction between the high resolution requirement of a WSI and the large color difference within a WSI, we propose a gradient-guided high-resolution stain migration network. The network is based on an unpaired adversarially generated network and does not require additional labeling to participate in training. We propose a new paradigm for pairing training to solve the possible problem caused by large color differences within a WSI. This paired training mechanism also ensures that we learn the correct staining without degrading the WSI resolution. Finally, we note that the edge gradients of the nucleus and cell membrane are large, so we guarantee that our stain migration network can keep the edge clear by using a gradient-guided loss function. In summary, our main contributions in this study are as follows:

(1) We propose a stain transfer network based on an unpaired adversary-generative network to solve the generalization problem encountered in the multi-center testing of benign and malignant diagnostic models. The network does not require additional data labeling and is easy to deploy.
(2) We propose a new pair-wise training paradigm to train our proposed generative network, which is able to learn the correct color transfer model at high resolution, solving the contradiction between the high resolution of a WSI and the large internal color difference.
(3) We introduce a gradient-guided loss function to train our proposed generative network, which can ensure the clarity of the generated images at the edges of the nucleus and cell membrane, which is helpful to improve the performance of the benign and malignant diagnostic model.

The content of this paper is arranged as follows: Section 2 introduces our proposed gradient-guided high-resolution dye migration network. We first give an overview of our proposed network, and then introduce the generator architecture, discriminator architecture, and gradient-guided loss function of the network, respectively. Section 3 experimentally verifies the effectiveness of our algorithm by first showing the visual differences between the pictures generated by our proposed network and the baseline method. We compare the color differences in the generated pictures at a larger scale and the differences in the details of the generated pictures at a smaller scale. We then tested the performance of our stain migration algorithm compared with the baseline method when tested in multiple centers through two centrally collected WSI datasets. Section 4 presents a discussion of the content of this paper, and Section 5 presents the conclusion of the text.

## 2. Materials and Methods

In this section, we first introduce the data and preprocessing method, and then elaborate on the model proposed in this paper. The proposed model is an improvement on the cycleGAN skeleton, which is mainly optimized on the high-resolution generator, discriminator, training strategy, and loss function. The following are explained in turn.

### 2.1. Dataset and Preprocessing

The TCGA-Lung dataset is a dataset for the diagnosis of benign and malignant lung cancer, which is formed by merging two public datasets, the TCGA-LUAD dataset [35] and TCGA-LUSC dataset [36]. Both TCGA-LUAD and TCGA-LUSC only contained a single lung cancer subtype, while the biggest challenge in WSI’s classification of benign and malignant lung cancer is that lung cancer has multiple subtypes. Therefore, we combined the two datasets to simulate the clinical classification of benign and malignant lung cancer. The combined data included two subtypes of lung cancer: adenocarcinoma and squamous cell carcinoma. A total of 1054 WSIs were included, of which we discarded four WSIs as they were low quality and randomly took out 210 WSIs for the independent test set.

After approval from the medical ethics committee, 1000 WSIs were independently collected for the classification of benign and malignant lung cancer. The WSIs included three lung cancer subtypes (adenocarcinoma, squamous cell carcinoma, and small cell carcinoma), more similar to the data distribution that may be encountered in clinical diagnosis. Since the data are collected internally, they are temporarily named Inhouse-Lung, which means internal data. These data contain a total of 694 malignant WSIs and 306 benign WSIs, so the data are unbalanced and difficult to classify. Similarly, we randomly selected 200 WSIs from them in proportion to malignancy and correspondence as an independent test set.

We used both datasets to simulate multi-center testing. For the TCGA-Lung dataset, we retained the original 210 WSIs as an independent test set, selected 100 WSIs from the remaining images for training the staining migration model only, and the remaining 740 WSIs for training the multiple instance learning (MIL) model. For the Inhouse-Lung dataset, the original 200 WSIs were also retained as an independent test set, 100 WSIs were randomly selected from the remaining 800 WSIs to train the stain migration model only, and the remaining 700 WSIs were used to train the MIL model. The specific experimental setup is shown in Table 1. In the data preprocessing, we used the preprocessing method to convert a WSI into a patch image; here, we set the size of the patch image to 512 × 512 pixels.

### 2.2. A High-Resolution Staining Style Transfer Model Based on Gradient Guidance

Due to the great achievements made by the Generative Adversarial Networks (GAN) model in the field of image generation, most of the existing style transformation methods are based on this model [37,38]. The pathological images of different patients have similarities in structural texture, such as the distribution of the nucleus and cytoplasm, and the distribution of the septum of the cells. However, due to the differences in staining agents and the differences in staining process, the distribution of pathological image data in color in different medical centers is not consistent. However, due to the similarity of image structure distribution, the color distribution of pathological images in different medical centers has a mapping relationship. Although data from different centers do not have paired images, there should be an underlying correspondence in the color distribution. Therefore, this paper chooses cycleGAN as the skeleton to train and learn this correspondence, and uses gradient guidance to select the pairing of data so that the model training is more stable.

**Figure 2:**
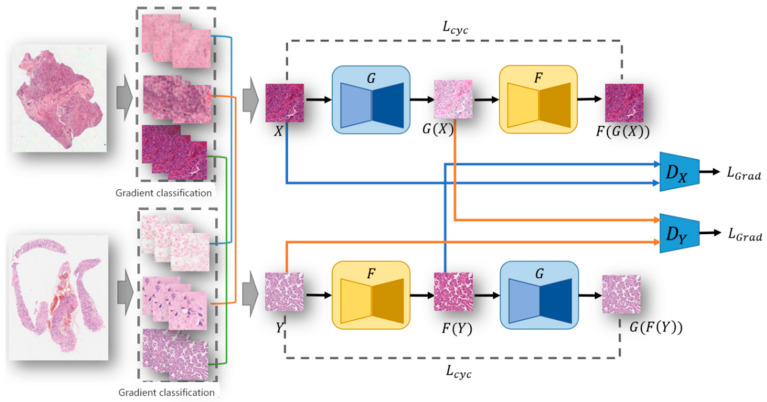
Overall framework for a gradient-guided high-resolution staining style transfer network.

Our proposed gradient-guided high-resolution dye migration network (HDGAN) is shown in Figure 2. The training process of the network is described as follows:

(1) Pretreatment. To preserve the resolution of the WSI after migration staining, we directly clipped the WSI into small block pictures using a sliding window at the resolution we wanted to preserve, which in our experiments was chosen at a magnification of 20×, which is routinely used in benign and malignant diagnostic models.
(2) The gradient density and classification were calculated. After cropping into small patch images, we use the Sobel operator to detect edges for each patch image and calculate the proportion of the number of pixels of the edge to the number of pixels of the whole patch image, which we call the gradient density. According to the gradient density, the patches were divided into three categories: gradient sparse (<10%), gradient medium (10–50%), and gradient dense (>50%).
(3) The pair constraint strategy was used to train the adversary-generative network. Simply put, when selecting the input images of the adversative generation network, it is necessary to pair them according to the gradient density. Only images belonging to the same class of gradient density can pair the input network for training. The reason for this is that combinations of pictures with the same density gradient tend to have the same tissue structure, which can avoid learning the staining style of other tissue structures during the process of staining migration.

In the test phase, in order to obtain the complete WSI images after staining and transfer, and to ensure the smooth color transition of WSI (that is, there is no obvious difference in the stitching of the two small images), we used the sliding window method to perform staining transfer on the complete WSI. Specifically, we used a sliding window to extract the small patch images of WSI and send them to the network to complete the color transfer. The sliding interval of the sliding window was selected as the general length of the small patch images, so that there would be overlap between two adjacent small patches, and the overlap was averaged.

#### 2.2.1. Unpaired Adversarial Generative Networks

**Figure 3:**
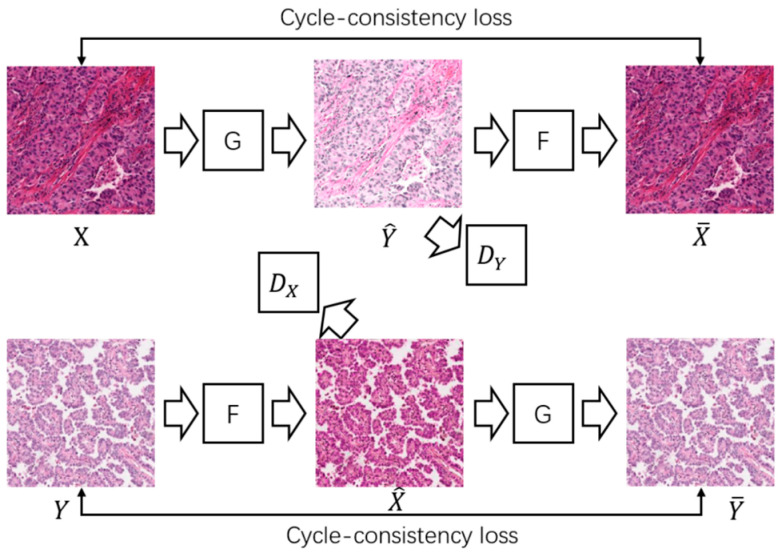
Network structure of CycleGAN.

The unpaired adversative generative network CycleGAN [29] and its variants are widely used in stain migration of digital pathological images [16,28,39]. The so-called unpaired means that the training data of the network does not need pixel-wise one-to-one correspondence, and only the image datasets of the two dyeing styles need to be collected separately. Figure 3 shows the network structure of CycleGAN, where X and Y are digital pathological images from two medical centers, respectively, G:X→Y and F:Y→X are generators, which are responsible for converting the picture of one center into the staining style of the other center, and $D_{X}$ and $D_{Y}$ are discriminators, which are used to determine whether the picture is real or generated.

The training of CycleGAN was accomplished by alternately training the generator and the discriminator. In the training generator stage, X obtains the stained transferred picture $\hat{Y}$ through generator G, and then reconstructs back to the original stained style picture $\hat{X}$ through generator F, and does the same for Y. CycleGAN requires that the reconstructed picture $\hat{X}$ be as close as possible to the original picture X, and similarly, $\hat{Y}$ should be as close as possible to Y. The above requirements are carried out by minimizing Cycle-consistency loss, which is usually the L1 norm loss function. At the same time, CycleGAN also requires that the generated pictures $\hat{X}$ and $\hat{Y}$ should be able to fool the discriminator, so they are given a label of 1 (that is, they are considered to be real), and the loss of both is calculated. In the training discriminator stage, CycleGAN requires the discriminator to be able to identify both real and generated pictures, so the generated pictures are given a label 0 and their loss is calculated.

#### 2.2.2. High-Resolution Image Conversion

Although CycleGAN achieves good results on unpaired image conversion, it often cannot be applied to high-resolution image conversion. Compared to CycleGAN, Pix2pixHD is trained on paired images, so it has more information for supervised training. Currently, Pix2pixHD can generate up to 2k resolution images and achieve good results in street view generation. Pix2pixHD uses a supervised training structure, so there is no need to constrain the reconstructed image to be consistent with the original image through Cycle-consistency loss. In Pix2pixHD, the authors used perceptual loss to constrain the generated image, which requires that the semantic information extracted using a deep neural network between the generated image and the target image is also consistent.

#### 2.2.3. Generator Network Architecture

**Figure 4:**
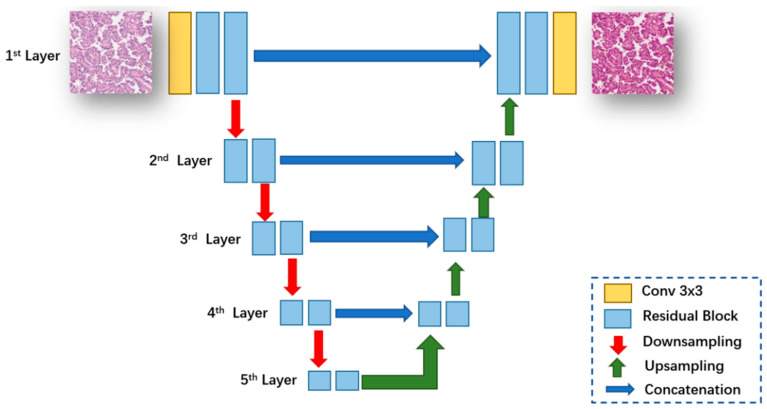
Generator network architecture.

We design our generator based on the U-Net [40] architecture, whose network architecture is shown in Figure 4. It consists of a contractile path (left side) and an expanding path (right side). The input image is first fed into a convolutional block to extract the low-level feature map. These features are then passed through the residual block [41] to extract a higher level feature map. The feature maps are then downsampled and fed to the next layer. The contraction and expansion paths follow the typical architecture of convolutional networks. Skip junctions [40] are applied to each layer to connect the features of each layer in the contraction patch with those in the expansion path.

#### 2.2.4. Discriminator Network Architecture

**Figure 5:**
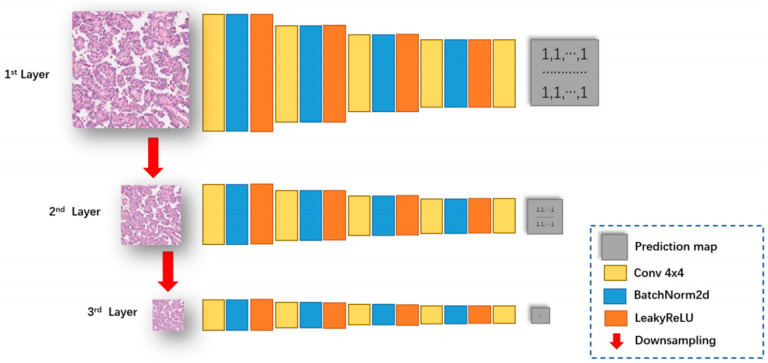
Discriminator network architecture.

In order to ensure that the pictures after dye transfer remain sufficiently clear, we designed a multiscale discriminator architecture to strengthen the discriminative ability of the discriminator, and its network architecture is shown in Figure 5. The input image is downsampled twice to obtain a total of three scales of images. The images of the three scales are judged by the convolutional neural network, and finally the discrimination matrix under the three scales is obtained. When calculating the discriminator loss, we will use the discriminant matrices at all three scales. We introduce the gradient-guided loss function GGGAN Loss [42] into the discriminator to ensure that the edges of the generated image are clear. We denote the input image as X and its corresponding gradient map as X’. Then, the input of layer 1 of the discriminator can be expressed as follows:

(1) $$X_{l}=[X_{\frac{1}{2^{\left(l-1\right)}}},(X_{\frac{1}{2^{\left(l-1\right)}}})\prime ],l\epsilon \{1,2,3\}$$

where $X_{\frac{1}{2^{\left(l-1\right)}}}$ represents X after downsampling by a factor of $2^{\left(l-1\right)}$. We let $m^{l}$ be the subsampled segmentation map of the l-th layer.

### 2.3. Paired Training Strategy

When WSI preprocesses small images, it needs to calculate the gradient density through a Sobel operator, and the images are divided into three categories according to the size of the gradient density. This was performed to meet the challenge of the large variation in staining styles of different tissue structures within the WSI. In WSI, a region with a large gradient density represents the presence of more nuclei in the region, whereas a small gradient density represents other structures in the pathological tissue. Therefore, by pairing training, the network can avoid learning the color style that is not related to the organization structure of the input picture during training. The paired training strategy can be viewed as a way to introduce prior knowledge into the model.

Our network architecture belongs to a variant of the unpaired adversative generative network CycleGAN. Therefore, in each iteration, we need to take a picture from the input set and the target set to form a pair of inputs for training, and the training process is used to so that the images in the pair can learn each other’s style. In our training strategy, we always require that the input image pairs come from the same class of gradient density when we select them, and the operation is the same as CycleGAN.

### 2.4. Gradient-Guided Loss Function

The edge information in digital pathology images is very critical for benign and malignant differentiation. Irregular cell membrane edges often indicate the possibility of cancer. Therefore, it is very important to ensure that the edge is clearly visible during the process of staining and migration. When selecting Cycle-consistency loss, the perception error makes up for the deficiency of L1 norm as the loss function to a certain extent, but the protection of the edge is not enough. Jiang et al. [42] introduced a gradient-guided loss function in the synthesis of mammograms to solve the problem of the disappearance of calcifications in the synthesized images. They explicitly calculated the gradient of the input picture using the Sobel operator and used it as an additional channel to participate in the discriminator for discrimination along with the generated picture.

We represent *X* as a digital pathology picture from one center, *Y* as a digital pathology picture from another center, *G:X→Y* and *F:Y→X* as generators, $D_{X}$ as a multiscale discriminator to distinguish real and synthetic WSI examples, and $D_{Y}$ as a multiscale discriminator to distinguish real and synthetic WSI examples. In addition, we denote that $\hat{X}=F(Y)$ and $\hat{Y}=G(X)$.

The loss function of the backpropagation discriminator $D_{X}$ can be expressed as

(2) $$L_{Grad}(D_{X})=\sum_{l=1}^{3}\left[{\left(D_{X}^{l}(X_{l})-m^{l}\right)}^{2}+{\left(D_{X}^{l}({\hat{X}}_{l})-m^{l}\right)}^{2}\right]$$

where $D_{X}^{l}$ represents the *l*-th layer of the multiscale discriminator $D_{X}$, ${\hat{X}}_{l}=[{\hat{X}}_{\frac{1}{2^{\left(l-1\right)}}},({\hat{X}}_{\frac{1}{2^{\left(l-1\right)}}})\prime ]$.

Similarly, we have

(3) $$L_{Grad}(D_{Y})=\sum_{l=1}^{3}\left[{\left(D_{Y}^{l}(Y_{l})-m^{l}\right)}^{2}+{\left(D_{Y}^{l}({\hat{Y}}_{l})-m^{l}\right)}^{2}\right]$$

where $D_{Y}^{l}$ represents the *l*-th layer of the multiscale discriminator $D_{Y}$, ${\hat{Y}}_{l}=[{\hat{Y}}_{\frac{1}{2^{\left(l-1\right)}}},({\hat{Y}}_{\frac{1}{2^{\left(l-1\right)}}})\prime ]$.

The loss function of the backpropagation generator G follows CycleGAN and can be expressed as follows:

(4) $$L(G)=L_{GAN}(D_{Y},\hat{Y})+\lambda L_{cyc}(G,F,X)$$

where λ is the hyperparameter used to balance $L_{GAN}$ and $L_{cyc}$.

(5) $$L_{GAN}(D_{Y},\hat{Y})=\sum_{l=1}^{3}{\left(D_{Y}^{l}({\hat{Y}}_{l})-m^{l}\right)}^{2}$$

(6) $$L_{cyc}(G,F,X)={\left\|F(G(X))-X\right\|}_{1}$$

Similarly, the loss function of the directional propagation generator F follows CycleGAN and can be expressed as follows:

(7) $$L(F)=L_{GAN}(D_{X},\hat{X})+\lambda L_{cyc}(F,G,Y)$$

where λ is the hyperparameter mentioned earlier.

(8) $$L_{GAN}(D_{X},\hat{X})=\sum_{l=1}^{3}{\left(D_{X}^{l}({\hat{X}}_{l})-m^{l}\right)}^{2}$$

(9) $$L_{cyc}(F,G,Y)={\left\|G(F(Y))-Y\right\|}_{1}$$

The training process of our proposed network is the same as that of CycleGAN: alternately training the generator and the discriminator, respectively, by alternating the two with backward error propagation.

### 2.5. Evaluation Metrics

The classification performance of MIL model was evaluated using the commonly used evaluation metrics in binary classification models, including accuracy, precision, recall, F1-score, and AUC.

Accuracy is used to evaluate the probability that the model prediction is correct across all samples.

(10) $$accuracy=\frac{TP+TN}{TP+FN+FP+FN}$$

Precision is used to evaluate the probability that a prediction is correct in a predicted positive sample.

(11) $$precision=\frac{TP}{TP+FP}$$

Recall is used to evaluate the probability that a positive sample can be found by the model.

(12) $$recall=\frac{TP}{TP+FN}$$

The F1-score is an index used to measure the accuracy of a binary classification model in statistics. It takes into account the precision and recall of the model at the same time. The F1-score can be regarded as the harmonic average of precision and recall.

(13) $$F1-score=\frac{2\times precision\times recall}{precision+recall}$$

Here, TP represents true positive, FP represents false positive, FN represents false negative, and TN represents true negative.

We obtained the area under the ROC curve (AUC) through calculation. The abscissa of the ROC curve is the false positive rate FPR, and the ordinate is the true positive rate TPR. By taking different thresholds for the prediction results, we can calculate the FPR and TPR under different thresholds. By connecting these coordinate points, we can obtain the ROC curve.

## 3. Results

This section first introduces the details of the experimental setup, and then shows the performance of the model with visual intuitive evaluation and quantitative evaluation, respectively. Since the image data from the different centers were not paired, a direct comparison could not be made. Therefore, we use the contrast of large-scale images and the contrast of small-scale images in visual intuitive evaluation, mainly comparing the color, contrast, texture structure, and other aspects of the image. Then, in comparison with quantitative metrics, we selected downstream classification tasks for experimental presentation. The color image style transformation is essentially used to achieve the normalization of different center data staining, so as to show the generalization performance of downstream tasks such as classification and segmentation models.

### 3.1. Implementation Details

In experiments to evaluate the dye migration effect of our proposed model, we used CycleGAN [29] as a baseline model of dye migration. In evaluating the dye migration network for improving the generalization performance of MIL models in multi-center tests, we selected the following MIL models: MI-Net [43], MIL-RNN [44], Att-MIL [4], CLAM [5], DHMIL [45], TSML-MIL [46], and IMIL [47]. The above MIL models all use ResNet18 [41] as the network architecture of the feature extractor, in which IMIL uses SimCLR [48] to initialize the feature extractor, and the maximum number of iterations is three. All the above experiments were performed on a single NVIDIA Quadro RTX 8000 GPU with 48,601 MB memory. In the training process of HDGAN, Adam [49] optimizer is used, the batch size is set to four, and the training is completed in a total of 60 h.

### 3.2. Visual Evaluation of Staining Migration Effect

We trained the staining migration model using 100 WSIs, each reserved in TCGA-Lung and Inhouse-Lung. We first evaluated the stain migration effect of HDGAN at large-scale. Note that large-scale digital pathology images were merged by using an overlapping sliding window approach and were not directly generated by the network, as described in the Section 2. The specific visual effects of digital pathology image synthesis and analysis are detailed in Section 4.

### 3.3. Evaluation of Generalization Performance

We first trained the MIL model on the 700 retained WSIs using Inhouse-Lung as the training center data. Then, the performance of the MIL model was tested using the independent test set (210 WSIs in total), retained in the TCGA-Lung as the test center. We then repeated the above experiments using TCGA-Lung as the training center data and Inhouse-Lung as the testing center. The experimental results are collated in Table 2 and Table 3, respectively. In this study, a *t* test was performed on all evaluation indicators, and the results showed that these differences were statistically significant (*p* < 0.05).

We used a total of three testing methods: 1. Direct testing. The TCGA-Lung images were directly used as input for testing. 2. Testing after CycleGAN staining and migration. CycleGAN was used to convert the TCGA-Lung images into the staining style of the training center, and then input them into the model for testing. 3. Testing performed after HDGAN staining and migration.

The experimental results are shown in Table 2 and Table 3.

(1) When the test data and the training data are not from the same center, the performance gap between MIL models is small. The AUC of all directly tested MIL models in Table 2 was about 0.7. It is lower in Table 3.
(2) After dyeing and migration, the performance of the model was improved, and HDGAN improved the performance of the model to a greater extent than CycleGAN.
(3) After staining and migration, the performance gap between MIL models began to appear. In Table 3, after HDGAN staining and migration, the AUC of IMIL was increased to 0.8313, while that of MI-Net was increased to 0.7233, and the AUC gap between the two was 0.1080.

## 4. Discussion

**Figure 6:**
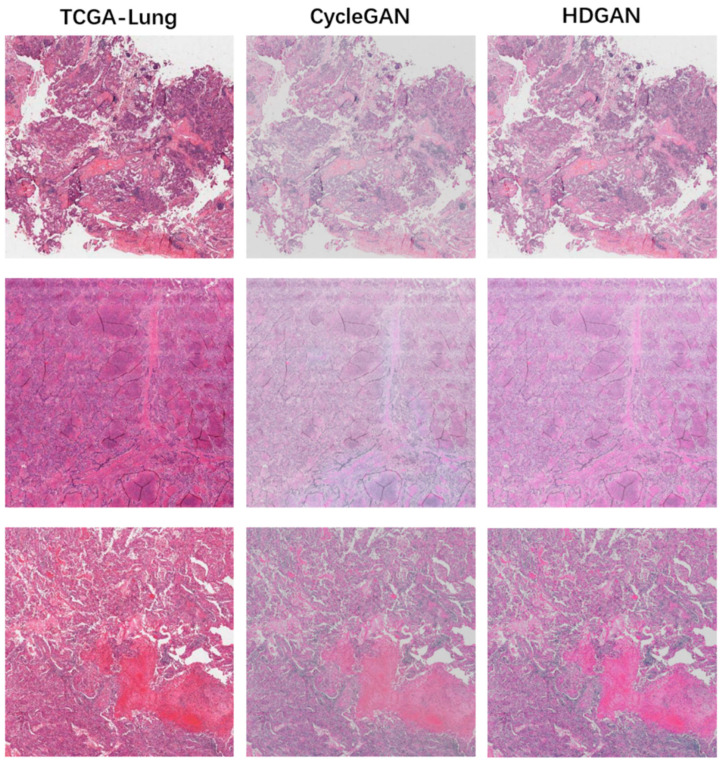
The evaluation of the stain migration effect at a large scale. The first column is from the digital pathological images of TCGA-Lung, the second column is the staining migration effect of CycleGAN, and the third column is the staining migration effect of our proposed method, HDGAN.

**Figure 7:**
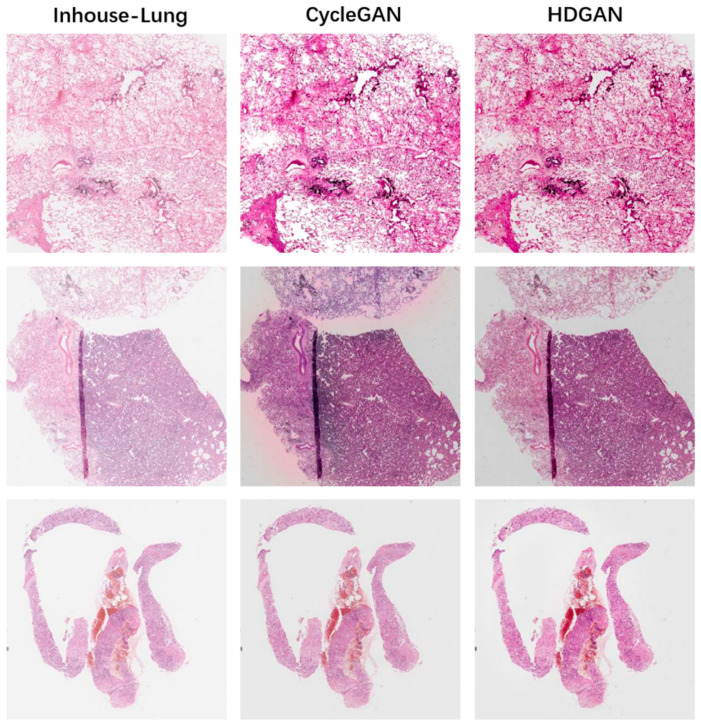
Assessing the effect of stain migration at large scales. The first column is from the digital pathology images of Inhouse-Lung, the second column is the staining migration effect of CycleGAN, and the third column is the staining migration effect of our proposed method, HDGAN.

We used the TCGA-Lung dataset and Inhouse-Lung dataset to simulate two medical centers. Figure 6 shows the staining migration results of TCGA-Lung, where the first column is the original figure in the dataset, and the second and third columns are the staining migration results of CycleGAN and HDGAN, respectively. We observe that at large scales, CycleGAN generates pictures with lower contrast and a preference for learning the average staining of the Inhouse-Lung. Figure 7 illustrates the results of stain migration for Inouse-Lung, which is visually clearer after dye migration due to the low contrast of the picture itself for this dataset. In general, after HDGAN is used to stain and transfer pathological images, the overall visual effect of the pictures is better, which is more conducive to clinical application. This is because the paired training strategy proposed by our model plays a role. If the traditional cycleGAN training strategy is adopted, that is, two images are randomly paired for training, the color contrast of the training result will be poor, because the color, chromaticity, and saturation of the digital pathological images in the same center are very different. We divided the dataset into multiple groups by calculating the gradient value of the images so that the images with dense cell distribution in the two central datasets were paired together, and the images with sparse cell distribution were assigned together. To a certain extent, this strategy makes the content information of the two images close in the training process, and makes the model pay more attention to the learning of staining features, thereby improving the performance of the model.

**Figure 8:**
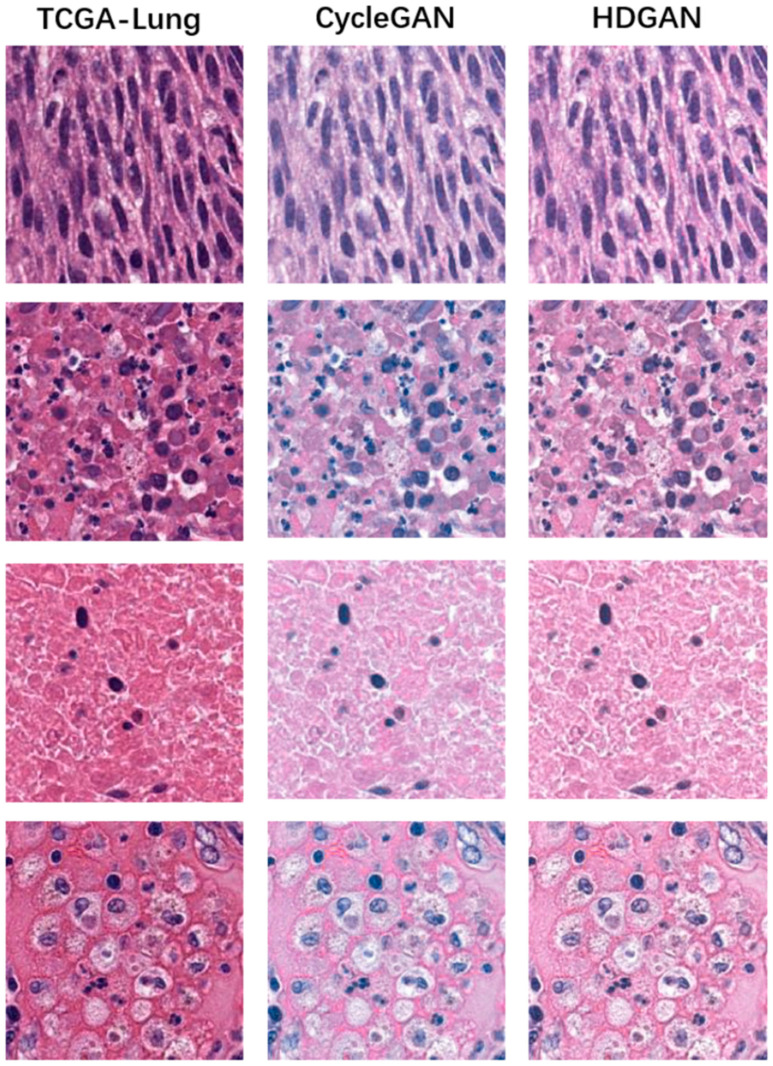
The evaluation of the stain migration effect at a small scale. The first column is from the digital pathological images of TCGA-Lung, the second column is the staining migration effect of CycleGAN, and the third column is the staining migration effect of our proposed method, HDGAN.

**Figure 9:**
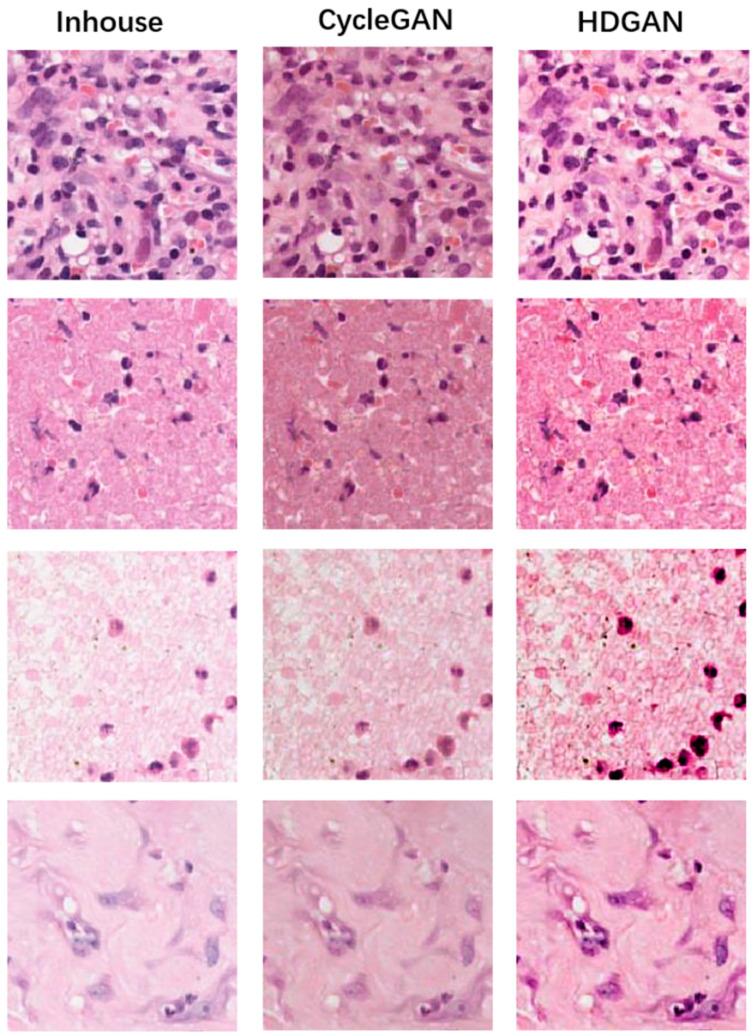
Assessing the effect of stain migration at a small scale. The first column is from the digital pathology images of Inhouse-Lung, the second column is the staining migration effect of CycleGAN, and the third column is the staining migration effect of our proposed method, HDGAN.

We next evaluated at small scales and, as above, Figure 8 and Figure 9 demonstrate the results of mutual staining migration between CGA-Lung and Inhouse-Lung, with image blocks in the figure all intercepted at 20× magnification. Similarly, we observed that CycleGAN generated images with lower contrast and at the same time less sharpness at the nuclear edges than HDGAN. Moreover, the HDGAN proposed in this paper has a more obvious contrast in its details and textures, so it will be more conducive to the MIL model for feature extraction, classification, and other operations. Since we use the gradient-guided loss function, the model will pay more attention to the edge gradient information of the generated image, making the edges and contour textures of the generated image clearer. The clear boundary information is also more conducive to the MIL model for feature extraction, classification, and other operations, and further improves the generalization ability of MIL model.

However, from the table of quantitative indicators, we can see that when the test data and the training data are not from the same center, the performance gap between the MIL models is small. Since the multi-center dataset is all about benign and malignant classification, there are no pathological images of healthy lungs in the data. Therefore, we examined the performance of the proposed model using the MIL model for benign and malignant classification. Here, DHMIL [43], TSML-MIL [44], and IMIL [45] are the pathological image classification models proposed by our previous study, which have achieved good results in the corresponding classification tasks. This indicates that none of the MIL models showed better generalization performance when the training and test sets were from different centers. Comparing the two tables, the performance of each MIL model was greatly improved after using the staining migration model. This suggests that the staining migration model is very important for the role of other downstream tasks.

In all directly tested MIL models in Table 1, the AUC was about 0.7; it is lower in Table 2, which should be related to the distribution of data in different centers and the amount of data. This also shows that the gap in the distribution of data from different centers is large, so the normalization operation of data from different centers is of great importance for segmentation models, classification models, and other algorithms directly applied in clinical practice. After dyeing and migration, it can be seen that the performance of the models has been improved, among which HDGAN improves the performance of the model to a greater extent than CycleGAN staining and migration. It shows that our model has some potential in the problem of multi-center data normalization. After staining migration was performed, the performance gap between MIL models began to emerge. In Table 2, after HDGAN staining and migration, the AUC of IMIL was increased to 0.8313, while that of MI-Net was increased to 0.7233, and the AUC gap between the two was 0.1080. Therefore, our method can be applied to different clinical application scenarios and can be adapted to different classification models, and is therefore valuable and has a prospect for clinical application.

## 5. Conclusions

In this study, we investigated how to improve the generalization ability of multi-example learning models without adding labeling information when faced with multi-centric data. In this paper, we propose an adversary-generative network-based style transfer algorithm for high-resolution images to ensure the generalization performance of the model on multi-center datasets. For a model trained on the target dataset, we wanted the source dataset to be transformed into the style of the target dataset by style transfer. We collected an equal number of target and source data, respectively, and used a sliding window to segment the WSI into small squares. The gradient density within the small squares was calculated by the gradient operator. We used an unpaired adversity-generation algorithm for the segmentation migration of the source data, but paired according to the gradient density at the time of input. At the same time, we introduced a gradient-enhanced regular term in the design of the loss function of the adversarial generation algorithm. The style transfer algorithm was verified in the lung cancer pathological image datasets of two centers. The performance of the domain-based multi-example learning model was significantly improved after using the style transfer algorithm on the source data. Our method can effectively improve the generalization ability of classification model on multi-center data. Therefore, our method can assist in improving the generalization performance of other task models based on pathological images from multi-center data. Clinically, our approach can improve the performance of downstream models on multi-center data, thereby improving the work efficiency of pathologists. In the future, we will try to extend the proposed method to more segmentation and classification tasks to verify the effectiveness of the proposed algorithm. In the future, we will try to extend this method to more segmentation and classification tasks to verify the effectiveness of the algorithm, and also try to collect more types of data and expand data samples to improve the generalization performance of the model.

## Figures and Tables

**Table 1:** Experimental settings.

	Train Set for Stain Migration Model	Train Set for MIL Model	Independent Test Set
TCGA-Lung	100	740	210
Inhouse-Lung	100	700	200

**Table 2:** The MIL model was trained with Inhouse-Lung as the training center dataset and tested on the independent test set of TCGA-Lung. The calculation of the *p*-value is based on the same method used in the Direct test for the control group, such as IMIL in cycleGAN and IMIL in the Direct test.

Method	MIL Model	AUC	*p*-Value	Accuracy	Precision	Recall	F1-Score
Direct test	MI-Net [43]	0.7012	-	0.7000	0.6750	0.7714	0.7200
	MIL-RNN [44]	0.6912	-	0.6810	0.6532	0.7714	0.7074
	Att-MIL [4]	0.7018	-	0.7000	0.6694	0.7905	0.7249
	CLAM [5]	0.7118	-	0.7095	0.6803	0.7905	0.7313
	DHMIL [45]	0.7018	-	0.7095	0.6803	0.7905	0.7313
	TSML-MIL [46]	0.7289	-	0.7095	0.6803	0.7905	0.7313
	IMIL [47]	0.7312	-	0.7095	0.6803	0.7905	0.7313
CycleGAN [28] used for stain migration before testing	MI-Net [43]	0.8502	<0.05	0.8381	0.8901	0.7714	0.8265
	MIL-RNN [44]	0.8622	<0.05	0.8476	0.9101	0.7714	0.8351
	Att-MIL [4]	0.8811	<0.05	0.8619	0.9419	0.7714	0.8482
	CLAM [5]	0.8817	<0.05	0.8714	0.9643	0.7714	0.8571
	DHMIL [45]	0.8835	<0.05	0.8714	0.9643	0.7714	0.8571
	TSML-MIL [46]	0.8856	<0.05	0.8714	0.9535	0.7810	0.8586
	IMIL [47]	0.8856	<0.05	0.8810	0.9762	0.7810	0.8677
HDGAN used for stain migration before testing	MI-Net [43]	0.8634	<0.05	0.8524	0.8627	0.8381	0.8502
	MIL-RNN [44]	0.8818	<0.05	0.8762	0.8990	0.8476	0.8725
	Att-MIL [4]	0.9011	<0.05	0.8905	0.9184	0.8571	0.8867
	CLAM [5]	0.9011	<0.05	0.8905	0.9184	0.8571	0.8867
	DHMIL [45]	0.9220	<0.05	0.9000	0.9286	0.8667	0.8966
	TSML-MIL [46]	0.9223	<0.05	0.9000	0.9286	0.8667	0.8966
	IMIL [47]	0.9243	<0.05	0.9048	0.9381	0.8667	0.9010

**Table 3:** The MIL model was trained using TCGA-Lung as the training center dataset and tested on the independent test set of Inhouse-Lung. The calculation of the *p*-value is based on the same method used in the Direct test for the control group, such as IMIL in cycleGAN and IMIL in the Direct test.

Method	MIL Model	AUC	*p*-Value	Accuracy	Precision	Recall	F1-Score
Direct test	MI-Net [43]	0.5288	-	0.6150	0.6646	0.8231	0.7354
	MIL-RNN [44]	0.5128	-	0.6100	0.6711	0.7846	0.7234
	Att-MIL [4]	0.5671	-	0.6250	0.6846	0.7846	0.7312
	CLAM [5]	0.5510	-	0.6300	0.6842	0.8000	0.7376
	DHMIL [45]	0.5423	-	0.6300	0.6842	0.8000	0.7376
	TSML-MIL [46]	0.5647	-	0.6300	0.6842	0.8000	0.7376
	IMIL [47]	0.5832	-	0.6300	0.6842	0.8000	0.7376
CycleGAN [28] used for stain migration before testing	MI-Net [43]	0.7189	<0.05	0.7500	0.7740	0.8692	0.8188
	MIL-RNN [44]	0.7191	<0.05	0.7400	0.7671	0.8615	0.8116
	Att-MIL [4]	0.7634	<0.05	0.8000	0.8358	0.8615	0.8485
	CLAM [5]	0.7658	<0.05	0.8000	0.8358	0.8615	0.8485
	DHMIL [45]	0.7823	<0.05	0.8000	0.8309	0.8692	0.8496
	TSML-MIL [46]	0.7923	<0.05	0.8200	0.8561	0.8692	0.8626
	IMIL [47]	0.8012	<0.05	0.8150	0.8496	0.8692	0.8593
HDGAN used for stain migration before testing	MI-Net [43]	0.7233	<0.05	0.7400	0.7635	0.8692	0.8129
	MIL-RNN [44]	0.7233	<0.05	0.7450	0.7651	0.8769	0.8172
	Att-MIL [4]	0.7787	<0.05	0.8100	0.8382	0.8769	0.8571
	CLAM [5]	0.7802	<0.05	0.8200	0.8507	0.8769	0.8636
	DHMIL [45]	0.8011	<0.05	0.8200	0.8507	0.8769	0.8636
	TSML-MIL [46]	0.8281	<0.05	0.8400	0.8769	0.8769	0.8769
	IMIL [47]	0.8313	<0.05	0.8400	0.8769	0.8769	0.8769

## Data Availability

Data cannot be shared publicly due to privacy protection of the participants and ethical restrictions. For researchers interested in the data, requests can be made to the corresponding author luyao23@mail.sysu.edu.cn. Researchers with questions about the model code covered in the article may also make a request to the corresponding author luyao23@mail.sysu.edu.cn.
